# Peripheral blood lymphocyte subset levels differ in patients with hepatocellular carcinoma

**DOI:** 10.18632/oncotarget.13041

**Published:** 2016-11-03

**Authors:** Hai-Zhou Liu, Wei Deng, Ji-Lin Li, Ya-Mei Tang, Li-Tu Zhang, Ying Cui, Xin-Qiang Liang

**Affiliations:** ^1^ Department of Medical Research, The Affiliated Tumor Hospital of Guangxi Medical University, 530021 Nanning, Guangxi, P.R. China

**Keywords:** hepatocellular carcinoma, CD3^+^, CD4^+^, CD19^+^, NK cells

## Abstract

We investigated the levels of target lymphocyte subsets in peripheral blood lymphocyte samples from patients with hepatocellular carcinoma (HCC). A total of 715 high-risk patients with primary HCC were recruited in Guangxi, China as the case group. The control group included 100 patients who received health examinations at the same hospital during the same period. Fasting elbow venous blood (10 mL) was collected from each participant, and flow cytometry was used to detect the levels of NK cells and CD3^+^, CD4^+^ and CD19^+^ T cells in peripheral blood samples. All included patients with prmary HCC were treated by surgical resection, and followed up for one year. The levels of CD19^+^ and NK cells were lower in cases than in controls (both *P* < 0.05). In addition, the level of CD8^+^ cells was greater in the case group than in the control group (*P* < 0.05). In the high-HCC-risk population, CD8^+^, CD19^+^ and NK cell levels all differed between male and female patients, patients in TNM stages I–II and stages III–IV, patients with and without extrahepatic metastasis, and patients with and without HBV infection (all *P* < 0.05). After follow-up, detected recurrence and survival rate was 33.71% and 83.64%, respectively. CD8^+^ levels was reduced following surgical resection, whereas the levels of CD19^+^ and NK cells were increased (all *P* < 0.05). In conclusion, altered levels of CD8^+^, CD19^+^ and NK cell levels may be used as reference values for monitoring immune function in certain populations with high HCC risk, and as potential evidence for the clinical diagnosis and treatment of HCC.

## INTRODUCTION

Hepatocellular carcinoma (HCC), usually secondary to either cirrhosis or viral hepatitis infection, is documented to be the fifth most common cancer in males (523,000 cases per year), the seventh most common cancer in females (226,000 cases per year) worldwide [1], and the third most common cause of cancer-related mortality globally [2]. The incidence rate of HCC varies substantially across different geographic regions, but the majority of the HCC burden is in developing countries, in which up to 85% of all cases take place [3]. Furthermore, over 75% of HCC cases occur in the Asia-Pacific region, and about half of HCC cases and deaths are estimated to occur in China [4, 5]. In China, there is a high-risk population for HCC, especially in the southeast coastal area of our country. Due to its asymptomatic features, HCC is often diagnosed at a late stage, at which point the disease is characterized by large tumors, unresectable lesions, a high metastasis rate, and a five-year survival rate below 5% [6, 7]. The etiology of HCC probably involves interactions among multiple risk factors, including nonspecific cirrhosis induced by chronic hepatitis B virus (HBV) or hepatitis C virus (HCV) infection, alcohol intoxication, exposure to aflatoxins, metabolic disorders, and immune-related dysfunction [8].

In the immune response to cancer, cellular and humoral immunity work together to kill tumor cells [9], but the former is more important than the latter. Lymphocytes are one of the most important cell groups in the immune system, and are divided into three categories based on the expression and biological function of their cell surface differentiation antigens: T lymphocytes (which are involved in cellular immunity), B lymphocytes (which are involved in humoral immunity) and natural killer (NK) cells (which directly kill tumor cells and virus-infected cells) [10–12]. Human tumor progression correlates significantly with immunologic function [13, 14]; for example, HBV infection disrupts the balance of the lymphocyte subsets and thereby changes their proportions in the peripheral blood, reflecting the immune state of body to a certain extent [15, 16].

T cells can be divided into cytotoxic T lymphocytes (CTL), helper T lymphocytes (TH) and suppressor T lymphocytes (TS) according to their functions and main surface markers (CD8, CD4, and CD19, respectively) [17]. CD8^+^ cells are also known as killer T cells; CD4^+^ cells can proliferate to activate other types of immune cells that produce direct immune responses or provide auxiliary help to other lymphocytes; and CD19^+^ cells inhibit helper T cell activity, thus indirectly inhibiting B cell differentiation and the TC killing function and negatively regulating the humoral and cellular immune responses of the T cell subsets [18, 19]. B cells participate in specific humoral immune responses; for instance, CD19^+^ B cells can only be activated and differentiate into mature plasma cells when there are TH cells and interleukin-1 regulated factors, to generate antigen-specific immunoglobulin and to have effect onhumoral immunity [20]. Natural killer (NK) cells depend on antigen stimulation, and can directly kill a variety of tumor cells and virus-infected cells (for instance, by activating phagocytic cells) [21].

In the present study, we detected the levels of the lymphocyte subsets in peripheral blood lymphocyte samples from HCC patients in China, in order to understand the immune status of these patients, and to obtain evidence about the changes in the proportions of these subsets for application in the clinical diagnosis and treatment of HCC.

## RESULTS

### General information and comparison of biochemical parameters

Basic information about the study subjects and the results of biochemical measurements for the study groups are shown in Table 1. In the case group (*n* = 715), there were 535 males and 180 females, with a mean age of 50.3 ± 10.3 years. In the control group, there were 83 males and 17 females, with an average age of 49.6 ± 10.5 years. There was no significant difference in gender or age between the groups (both *P* < 0.05). The body mass index (BMI) level was significantly greater in the case group than in the control group (*P* < 0.05). With regard to the tumor numbers in the HCC case group, 306 patients had a single tumor, while the remaining 409 patients had multiple tumors.

**Table 1:** General information and biochemical parameters comparison among the three different groups

	Case group (*n*= 715)	Control group (*n*= 100)	*P* value
Age (years)	50.3 ± 10.3	49.6 ± 10.5	0.526
Genders (M/F)	535/180	63/37	0.070
BMI	28.8 ± 5.1*	22.1 ± 4.4	< 0.001
Tumor sizes	5.5 ± 4.2	/	/
Tumor numbers (Singly/Multiply)	306/409	/	/
TNM stages (I/II/III/IV)	98/290/192/135	/	/
Child-Pugh (A/B/C)	458/139/118	/	/
Extrahepatic metastasis	139/576	/	/
HBV infection	574	19	< 0.001
HCV infection	141	0	< 0.001

Note:

* compared with the control group, *P* < 0.05. M, male; F, female; BMI, body mass index; HBV, hepatitis B virus; HCV, hepatitis C virus.

### Comparison of lymphocyte subset levels between groups

As shown in Table 2, the levels of NK cells and CD3^+^, CD4^+^, CD19^+^ T cells in peripheral blood samples all tended to be lower in the case group than in the control group; however, only the CD19^+^ and NK cell levels differed significantly between the groups (both *P* < 0.05). On the other hand, the level of CD8^+^ cells was clearly greater in the case group than in the control group (*P* < 0.05).

**Table 2:** The comparison of levels of lymphocyte subsets and NK cells between the case group and the control group

	*n*	CD8^+^	CD19^+^	CD3^+^	CD4^+^	NK
Case group	715	29.3 ± 11.6*	3.0 ± 2.7*	69.3 ± 11.9	35.1 ± 10.2	15.1 ± 8.3*
Control group	100	24.7 ± 6.1	13.6 ± 3.9	69.6 ± 10.5	35.7 ± 7.3	17.0 ± 5.2
*P* value		< 0.001	< 0.001	0.798	0.578	0.026

Note:

* compared with the control group, *P* < 0.05. NK, natural killer.

### Comparison of lymphocyte subset levels within the case group

The comparisons of CD8^+^, CD19^+^ and NK cell levels in patients with different clinical parameters within the case group are shown in Table 3. Within the high-HCC-risk population, significant differences in CD8^+^, CD19^+^ and NK cell levels were found between male and female patients, patients in TNM stages I-II and stages III-IV, patients with and without extrahepatic metastasis, and patients with and without HBV infection (all *P* < 0.05). However, the CD8^+^, CD19^+^ and NK cell levels did not differ significantly according to the tumor number or Child-Pugh distribution in this population (all *P* > 0.05).

**Table 3:** Lymphocyte subsets and NK cells levels comparison within the case group

	CD8^+^	CD19^+^	NK
Genders			
Male	30.1 ± 10.2*	3.0 ± 2.6*	16.1 ± 8.4*
Female	25.9 ± 9.7	3.3 ± 2.4	13.6 ± 8.0
Tumor numbers			
Singly	28.9 ± 10.1	3.0 ± 2.6	15.6 ± 8.3
Multiply	29.2 ± 10.2	3.0 ± 2.6	15.8 ± 8.3
TNM stages			
I–II	25.6 ± 9.8	3.3 ± 2.4	13.5 ± 8.0
III–IV	30.1 ± 10.1*	2.9 ± 2.7*	16.3 ± 8.3*
Child-Pugh			
A	27.5 ± 9.7	3.1 ± 2.5	14.8 ± 7.8
B	28.7 ± 10.0	3.0 ± 2.6	14.3 ± 8.0
C	29.0 ± 10.0	3.1 ± 2.6	15.6 ± 8.1
Extrahepatic metastasis			
With	30.1 ± 9.7*	3.1 ± 2.6*	15.9 ± 8.2*
Without	25.5 ± 9.4	3.3 ± 2.7	11.8 ± 7.7
Infection			
HBV infection	29.3 ± 10.2*	3.0 ± 2.6*	16.0 ± 8.4*
HCV infection	26.1 ± 9.5	3.3 ± 2.9	12.3 ± 8.0

Note:

* compared between groups, *P* < 0.05. Hepatitis B virus; HCV, hepatitis C virus.

### Survival analysis

Until January 2015, 38 cases of HCC patients were lost during the follow-up period, with a loss ratio of follow-up of 5.31%. The patients underwent surgical treatment, and the surgical treatment was good. There were no death and no complications (such as hepatic failure, hemorrhoea or abdominal infection) during treatment. Eligible patients were followed up for one year, 241 patients experienced tumor recurrence, and corresponding recurrence rate was 33.71%. Furthermore, after follow-up, there were 598 cases of patients survived from surgical treatment, with a survival rate of 83.64%. Accordingly, CD8^+^ levels was estimated to be reduced following surgical resection (29.3 ± 11.6 vs. 22.4 ± 8.9), whereas the levels of CD19^+^ (3.0 ± 2.7 vs. 11.4 ± 3.8) and NK cells (15.1 ± 8.3 vs. 17.8 ± 6.4) were on the other hand increased significantly, showing statistical difference (all *P* < 0.05).

## DISCUSSION

It is widely accepted that malignant tumors are caused by reduced immune function [13, 22]. Furthermore, there is ample evidence that the occurrence and development of HCC is associated with the status of the adaptive immune system, and that a normally functioning system can potentially induce therapeutic antitumor immunity [23, 24]. Lymphocytes (T lymphocytes, B lymphocytes and NK cells) are the most important cell group involved in maintaining immunological balance by suppressing a large number of immune responses [25–28]. Abnormal proportions of these subsets and their associated functions may disrupt the balance of the immune system [29]. In view of the important activities of these cells, we detected the levels of the different lymphocyte subsets in peripheral blood lymphocyte samples from patients with HCC.

We found that the levels of CD3^+^, CD4^+^, CD19^+^ T cells and NK cells in peripheral blood samples all tended to be reduced in high-risk HCC patients (the case group), whereas the level of CD8^+^ cells was greater in the case group than in the control group, suggesting that HBV infection alters the proportions of the peripheral blood lymphocyte subsets. A large number of studies have confirmed that T cells are one of the most important antiviral cell populations [30–32]. During both HBV and HCV infections, specific T cells are functionally depleted as they clear the infection [33]. Therefore, the reduced number of CD3^+^, CD4^+^, CD19^+^ T cells in patients with HCC to some extent indicates a decline in the body's antiviral ability, which ultimately contributes to the deterioration of diseases caused by persistent virus infection.

Furthermore, NK cells exert antiviral, antifibrotic and antitumor functions [18, 34, 35], and the reduced proportion of NK cells in patients with different stages of HBV infection suggests that NK cells may be involved in the progression of HCC. Previous work has shown that NK cells can kill hepatic stellate cells (HSCs), which are considered to be critical for the development of liver fibrosis [36]. High expression of HSCs activates α-smooth muscle actin, which induces the secretion of a large amount of collagen and ultimately leads to fibrosis and cirrhosis of the liver [37]. A variety of immune cells, cytokines, and chemokines in the host immune system have been suggested to stimulate the activation of HSCs in the development of hepatic fibrosis [38–40]. NK cells can suppress activated HSCs to inhibit hepatic fibrosis and further canceration, the mechanism of which may involve several molecular pathways (e.g., TRAIL, FAS/FASL and NKG2D) and requires further confirmation[41–43]. Consistent with these observations, NK cells tended to be reduced in high-risk HCC patients in this study, so the levels of these cells may be meaningful for predicting HCC progression.

Further investigation revealed that the proportions of the target lymphocyte subsets also differed significantly between male and female patients, patients in TNM stages I–II and stages III–IV, patients with and without extrahepatic metastasis, and patients with and without HBV infection within the high-HCC-risk population. However, the proportions of these subsets did not differ significantly according to the tumor number or Child-Pugh distribution, which may be attributed to the functions of the CD8^+^, CD19^+^ and NK cell levels in the high-HCC-risk population, and differences in the lifestyles, dietary patterns, health and economic conditions of the patients. The different lifestyles, dietary patterns, and health conditions between men and women may be critical determinants of the development of HCC. In addition, patients with aggressive clinicopathological features are obviously prone to serious disease progression; accordingly, TNM stage, metastasis, and HBV infection strongly correlated with the severity and extent of malignancy.

Meanwhile, as for the results of survival analysis after surgical treatment, corresponding recurrence rates was still relatively higher and the survival rates should be improved, level of CD8^+^ cells was reduced whereas CD19^+^ and NK cells leves were increased. Notably, the immune function of the body has a very complex regulatory mechanism, especially of the cellular immunity, all kinds of cell subsets and their products interact and regulate each other to maintain the normal immune response capability. Above mentioned lymphocyte levels changed significantly before and after treatment, suggesting that suitable therapies should be adopted to restore the immune state of the body as soon as possible, which will in turn contribute a lot to the reduction of complications and to promote postoperative recovery of disease courses, and thereby is beneficial for inhibiting *in vivo* rapid growth of metastasis caused by further decline in immune function.

To summarize, altered CD8^+^, CD19^+^ and NK cell levels may be used as reference values for monitoring the immune function of certain populations with high HCC risk, and as evidence for the clinical diagnosis and treatment of HCC. However, there were some limitations to this study. For instance, CD values are known to differ according to ethnicity, but only an area demonstrating high HCC risk in China was investigated in this study. Also, there are limited data regarding the association of CD values and NK cell levels with prognostic outcomes of HCC. Considering the above limitations, further studies should be conducted with larger sample sizes to validate the findings we have presented, analyze specific subgroups (such as patients after surgical intervention), and correlate these findings with post-surgery outcomes after long-term follow-up.

## MATERIALS AND METHODS

### Study objects

In total, 715 patients with high-risk primary HCC were recruited in Guangxi, China as the case group for the present study. These patients had been admitted to The Affiliated Tumor Hospital of Guangxi Medical University for surgical treatment from January 2012 to January 2014, and included 535 males and 180 females ranging from 26 to 78 years in age. All patients were scanned by means of magnetic resonance imaging, abdominal B ultrasound and computed tomography, and were examined for clinical symptoms and signs, with alpha fetoprotein levels of 17–3000 ng/mL. The inclusion criteria were: (1) confirmation of primary HCC by clinical manifestation and histopathological examination of liver biopsy specimens associated with imaging diagnosis; (2) diagnosis of HBV infection following serologic tests (HBsAg positive); (3) poor liver function or a tumor with a maximum diameter greater than 5 cm; and (4) no previous chemotherapy or radiation therapy. The exclusion criteria were: (1) severe dysfunction of the heart, liver or kidney; (2) combined immune and endocrine system diseases; (3) history of malignant tumors in other organs and liver metastasis; (4) antiviral drug treatment prior to admission; (5) concurrent HCV, hepatitis A, hepatitis E, or human immunodeficiency virus infection; and (6) other types of liver disease, such as autoimmune liver disease, inherited metabolic liver disease, alcoholic liver disease, schistosomiasis-related liver disease, liver disease of unknown cause, etc. The control group included 100 patients from The Affiliated Tumor Hospital of Guangxi Medical University who had received health examinations during the same period, among whom 63 were male, 37 were female, and the average age was 49.6 ± 10.5 years. The included controls were all negative for hepatitis B surface antigen/antibody, hepatitis B e antigen/e antibody, and hepatitis B core antibody, and had normal levels of alanine aminotransferase (ALT) and glutamic oxaloacetic transaminase (AST). This study was approved by the Ethics Committee of The Affiliated Tumor Hospital of Guangxi Medical University, and all the included subjects were informed of the study procedures and signed informed consent forms.

### Blood sample collection

Fasting elbow venous blood (10 mL) was collected from each participant in the morning, of which 5 mL was collected without an anticoagulant. These 5-mL samples were placed in a water bath at 37°C for 30 min and centrifuged at 1400 × g. Then, the supernatants were collected and kept at −20°C for the measurement of liver function indices on a HITACHI 7060 automatic biochemical analyzer (Hitachi 7060, Hitachi Ltd., Tokyo, Japan). The other 5 mL of blood was collected under general anticoagulation (into blood collection tubes containing EDTA-K3), and the specimens were prepared for flow cytometry within 30 min. To ensure maximum viability, stained cells were analyzed promptly (within 6 hours) for the detection of lymphocyte subsets.

### Establishment of the flow cytometry assay

First, cell staining was conducted according to the instructions for operation, and the antibodies (against CD8, CD19, CD3, CD4, and NK cells) were added directly to the bottom of each test tube. Second, 100 μL of evenly mixed whole blood was added directly to the bottom of the tube, and the tube was gently shaken. The flow tubes were protected from light and refrigerated at 4°C for 30 minutes so that the various antibodies could fully combine with their corresponding receptors. Then, red blood cell lysis buffer was added and the tubes were gently shaken to lyse the red blood cells in the samples, and the samples were allowed to stand for 10 minutes, protected from light. Subsequently, 1.5 mL PBS was added to terminate red blood cell lysis, and the test tubes were placed in an oscillator for gentle shaking. Samples were separated by means of an automatic balance centrifuge at 350 × g for 5 min. Then, the flow tubes were removed and the supernatants were quickly discarded so that only the bottom of the sediment was retained. Following the addition of another 2 mL PBS and gentle shaking, further centrifugation was conducted at 350 × g for 5 min, and the supernatants were quickly discarded while the sediment was retained. Then, 0.5 mL cell suspension was added for cell suspension and surface-labeled cell fixing, and the samples were placed at room temperature for 20 min, followed by another centrifugation at 350 × g for 5 min. After the supernatants were discarded, 2 mL film-breaking agent was added and a 10-min centrifugation was performed at 350 g/min. Furthermore, an intracellular labeled monoclonal antibody was added and mixed at 4°C for 30 minutes. Then, the samples were washed twice with film-breaking agent and centrifuged, the supernatants were discarded, and another 0.5 mL PBS was added for cell suspension. Flow cytometry (BD Biosciences, Franklin Lakes, NJ, USA) was performed to detect labeled cells and analyze the results.

### Follow-up

All included patients with prmary HCC were treated by surgical resection. Through telephone and outpatient follow-up, the follow-up period was lasted for one year until January 2015. Patients’ survival and recurrence rates were recorded in details during the follow-up period.

### Statistical methods

For statistical analysis, SPSS 21.0 statistical software (SPSS Inc, Chicago, IL, USA) was used in this study. Measurement data are represented as $\bar{x}\pm s$ and were validated by a normality test. Comparisons of measurement data were performed with the *t*-test, and comparisons of categorical data were performed with the chi-square test. Survival estimates were calculated by the Kaplan-Meier analysis. *P* < 0.05 was chosen as the significance level.
